# Obituary

**Published:** 1884-01

**Authors:** 


### Obituary.

---

### JAMES MARION SIMS, M.D.

The death of Dr. J. Marion Sims carries profound grief to the American profession. Not only in this country, but in whatever land true medicine lives, his departure will be mourned, for his fame and the influence of his teaching were almost world-wide. Sitting in the darkness of this sudden and great sorrow, it is natural to consider the work that he did in his busy life.

As there are epochs in the history of medicine with which famous names are associated, so there are famous names belonging to the special departments of medicine,—names of those who have founded or greatly advanced those special departments. As the circulation of the blood suggests the name of Harvey, or pathological anatomy that of Morgagni, or obstetric anæsthesia that of Simpson; as vaccination always brings up the name of Jenner, or auscultation that of Laennec,—so does gynæcology suggest that of Marion Sims; patient, brave, of restless activity; a triumphant, a noble man. He was a bold pioneer, opening new pathways; original and with creative genius, he discovered for himself, and made his discoveries a gift to the profession. Even when unconsciously repeating what had been done before him, he greatly enriched the discovery and carried the advance far beyond any predecessor. No matter what may be the great glory of those who have followed his footsteps, the best of them feel that they would never have accomplished what they have, had he not shown the way.

It is needless to say that before he faithfully worked out a method, the curing of genito-urinary fistulae in the female was a rare exception, and now, thanks to his patient, self-denying labors, it is the rule. The best method of making uterine examinations he discovered and taught. He has done more than any man of his generation to teach not only the diagnosis, but also the most important surgical therapeutics of uterine diseases. Going to New York after many a hard lesson, after working out many a difficult problem unaided in his Southern home, and thus made strong in self-reliance, he gave an impetus to the study of the diseases of women which must be felt long after his body mingles with kindred earth. The Women's Hospital of New York, which owes its foundation to him, has been a school of the most valuable instruction to the profession; and that hospital has led to the establishment of similar institutions in various parts of the country, blessing them who are relieved in each, and blessing the profession, to whom they have been great centers of light. His treatise upon uterine surgery is one of the master-pieces of medical literature of the country. It has been translated into several foreign languages, and is found wherever there are educated physicians.

It would require much space to give even a list of the instruments Dr. Sims has invented for the diagnosis or treatment of uterine diseases. Many of these are found in the consulting rooms of all gynæcologists, and in all hospitals where the diseases of women are treated. If to-day all the publications of his pen and all the instruments of his device were destroyed, a great blank in gynæcological science and art would be made,—a blank that would remain for years.

He was a man constantly trying to add to his knowledge in his special department, and how cheerful and helpful to all who sought his advice! Of great personal magnetism, he was an inspiration to all brought in contact with him. But his inspiring power was stronger, wider than this—it was far reaching, long-lasting, so that men who never saw him, felt his influence, and were quickened through all their lives by his example and printed words; hearts as well as heads acknowledged his power.

Marion Sims, in many respects the greatest gynæcologist of the century, has passed away, but he has left an undying fame. —*Medical News*, Nov. 17, 1833.

